# Health economic evaluations based on routine data in Germany: a systematic review

**DOI:** 10.1186/s12913-018-3080-3

**Published:** 2018-04-10

**Authors:** Fabia Mareike Gansen

**Affiliations:** Department of Health Care Management, Institute of Public Health and Nursing Research, Health Sciences, University of Bremen, Grazer Str. 2a, 28359 Bremen, Germany

**Keywords:** Economic evaluation, Cost-consequences analysis, Cost-effectiveness analysis, Routine data, Administrative data, Germany

## Abstract

**Background:**

Improved data access and funding for health services research have promoted the application of routine data to measure costs and effects of interventions within the German health care system. Following the trend towards real world evidence, this review aims to evaluate the status and quality of health economic evaluations based on routine data in Germany.

**Methods:**

To identify relevant economic evaluations, a systematic literature search in the databases PubMed and EMBASE was complemented by a manual search. The included studies had to be full economic evaluations using German routine data to measure either costs, effects, or both. Study characteristics were assessed with a structured template. Additionally, the Consolidated Health Economic Evaluation Reporting Standards (CHEERS) were used to measure quality of reporting.

**Results:**

In total, 912 records were identified and 35 studies were included in the further analysis. The majority of these studies was published in the past 5 years (*n* = 27, 77.1%) and used insurance claims data as a source of routine data (*n* = 30, 85.7%). The most common method used for handling selection bias was propensity score matching. With regard to the reporting quality, 42.9% (*n* = 15) of the studies satisfied at least 80% of the criteria on the CHEERS checklist.

**Conclusions:**

This review confirms that routine data has become an increasingly common data source for health economic evaluations in Germany. While most studies addressed the application of routine data, this analysis reveals deficits in considering methodological particularities and in reporting quality of economic evaluations based on routine data. Nevertheless, this review demonstrates the overall potential of routine data for economic evaluations.

**Electronic supplementary material:**

The online version of this article (10.1186/s12913-018-3080-3) contains supplementary material, which is available to authorized users.

## Background

Over the past few years, routine data has become an increasingly significant data source for health research in Germany [1]. In part, this development can be ascribed to improved access to routine data for research purposes after legal changes. Since 2011, the law “Versorgungsstrukturgesetz” provides the legal basis for a better database and easier utilization of health insurance claims data. In consequence, the German Institute of Medical Documentation and Information (DIMDI) has established a database consisting of pseudonymized claims data, which has been assessable for selected stakeholders since February 2014. In addition to these developments, the innovation fund (“Innovationsfonds”) of the Federal Joint Committee (G-BA) has encouraged the evaluation of health services including routine data analysis. From 2016 to 2019, associated funding of health services research will amount to 300 million euros. The improved data access and funding will likely lead to an increased research interest in the use of routine data for health economic evaluations. As a result, there is a need for a systematic overview of both the existing evidence and possible methodological deficits. To health services researchers, this review provides orientation for future research with the aim of improving research quality. For practitioners and policy makers, it is meant to give insights into the reliability of current studies and demonstrate the distinctive features of economic evaluations based on routine data.

This review builds upon previous research on the use of routine data in Germany. Topics covered up to date vary from single studies applying routine data as a data source [2, 3], methodological studies on its potentials and challenges [4–6] to reviews on the status and perspectives in health services research [1, 7]. In contrast to the examples above, the focus of this review is placed on the application of routine data for health economic evaluations analyzing costs as well as effects of health care interventions. In the context of the German statutory health insurance (SHI) system, which covers around 85% of the German population, health economic evaluations can be used to support reimbursement decisions. While most economic evaluations are conducted without the G-BA commissioning the Institute for Quality and Efficiency in Health Care (IQWiG), its annually updated General Methods paper provides guidelines for health economic research [8]. German industry stakeholders such as SHI funds or pharmaceutical companies commonly perform economic evaluations to investigate new interventions either on a voluntary or mandatory basis. As in the rest of Europe, economic evaluations in Germany have traditionally been based on primary data derived from clinical studies such as randomized controlled trials [9]. Routine data as an alternative data source is defined as electronically documented information which is generated in the process of administration, provision of services or reimbursement [10]. While it is not produced for research purposes, it can be used for such. In health services research, the most common form of routine data is claims data from health insurances. This review is based on a 2012 overview from Schreyögg and Stargardt [9] discussing the use of claims data for health economic research. It is meant to update and complement their findings on economic evaluations applying routine data in Germany.

Consequently, a main research objective of this review is to identify and characterize full economic evaluations based on German routine data. The analysis refers primarily to developments in the number and types of economic evaluations as well as the kind and use of routine data. An additional emphasis lies on the methodological specifics of routine data analysis such as addressing selection bias. Finally, this review aims to measure the reporting quality of health economic evaluations based on routine data.

## Methods

### Data sources and search strategies

The literature search and analysis performed are based on the Preferred Reporting Items for Systematic Reviews and Meta-Analyses (PRISMA) [11]. As a first step, a systematic search was conducted in the databases PubMed and EMBASE. The choice of these databases was made after a preliminary search in the DIMDI database. To identify relevant sources for the research question at hand, the terms “claims data”, “German” and “cost?” were searched connected by the Boolean operator AND. PubMed and EMBASE were among the databases with the most search results for the above search terms. Although the DIMDI database does not list all potentially applicable databases, PubMed and EMBASE were chosen as the main sources for this review. To conduct the literature search, a uniform search strategy was developed and adapted for each database. This search strategy included three search components. The first component was meant to identify studies using routine data, the next studies with a German setting and the last was to detect economic evaluations. The routine data component was based on related reviews [1, 7] while the component for economic evaluations consisted of a simplified version of the search algorithm used for the National Health Service Economic Evaluation Database (NHS EED) [12]. The exact search strategies are shown in Appendix 1. The first and third search component were limited to title, abstract and keywords using the parenthesized term *text word* in PubMed and *ti,ab,kw* in EMBASE. The search was performed on December 15, 2016. Additionally, a manual search was conducted among others in the database “Versorgungsforschung Deutschland” (health services research Germany) in the category “Gesundheitsökonomie” (health economics).

### Inclusion and exclusion criteria

To identify relevant studies, the following inclusion criteria were used: *(1)* A considered study had to be a full health economic evaluation defined by Drummond et al. as a “comparative analysis of alternative courses of action in terms of both their costs and consequences” [13]. Consequently, cost-consequences analyses, cost-effectiveness analyses, cost-utility analyses and cost-benefit analyses were included. Cost of illness and cost-minimization analyses were excluded to allow the analysis of effectiveness measures based on routine data. *(2)* To be included, a study had to use routine data as a data source to determine costs, effects or both costs and effects of an intervention. This application of routine data had to be stated in the study’s full text. The underlying definition of the term routine data is information generated in the process of administration, provision of services or reimbursement which is documented electronically [10]. A common example of routine data in health care is claims data from the statutory health insurance. To assess the diversity of routine data analysis, other sources of routine data were included in this review. Methodological publications on the use of routine data for health services research or related topics in health economics were excluded. *(3)* Relevant studies had to be based on German data only as this was the setting of the research objective at hand. Studies including data from other countries – such as comparative analysis – were not considered in this review. *(4)* Only original empirical studies presented as full text articles were evaluated. Methodological publications on routine data application were disregarded. Study protocols not reporting results or literature reviews as well as records presented only as abstracts, conference proceedings, letters, editorials, commentaries, posters or presentations were also excluded from further analysis. No limitation with regard to the publication date, language, study population, or intervention was applied.

### Review process

The review process involved screening all titles and abstracts which resulted from the databases and manual search according to the eligibility criteria. Relevant full text articles were retrieved and included in the further analysis. If title and abstract did not provide sufficient information on the inclusion and exclusion criteria, the full text of a record was retrieved and examined. The screening of titles, abstracts and relevant full texts was performed independently by two researchers. Disagreements between the two reviewers were settled through consensus or consultation of a third reviewer (for further information, see acknowledgments).

Further analysis was performed by filling out a standardized extraction form for all included studies. This template was based on a review by Rovithis [14] and consisted of three categories: general information, study design, and analytical approach. The first category contained bibliographic information, addressed disease, details on the intervention as well as funding. Details on the study design included the type of economic evaluation, source and use of routine data, study size, perspective, time frame, outcomes, costs, and summary measure. The evaluation of analytical approaches addressed methods for handling selection bias, data linkage, performance of uncertainty analysis and applied software. Where plausible, the extracted data for each item was categorized. According to the objectives of this review, a special focus was placed on the use of routine data and its methodological particularities. This was implemented by integrating selected items of the RECORD (REporting of studies Conducted using Observational Routinely-collected health Data) statement [15] into the evaluation of analytical approaches. Additionally, the quality of reporting was assessed based on the Consolidated Health Economic Evaluation Reporting Standards (CHEERS) statement [16, 17]. It consists of a 24 item checklist subdivided into the six categories title and abstract, introduction, methods, results, discussion and other. The checklist describes the minimum amount of information which should be provided in each category when reporting economic evaluations. Each of these items was assessed for every study using *yes* if the required information was reported, *no* if not and *n/a* if the item was not applicable. The studies were then evaluated depending on the percentage and type of criteria met. A benchmark of 80% fulfillment of applicable criteria was set to divide the identified studies into two groups according to the quality of their reporting.

## Results

### Search results

**Fig. 1:**
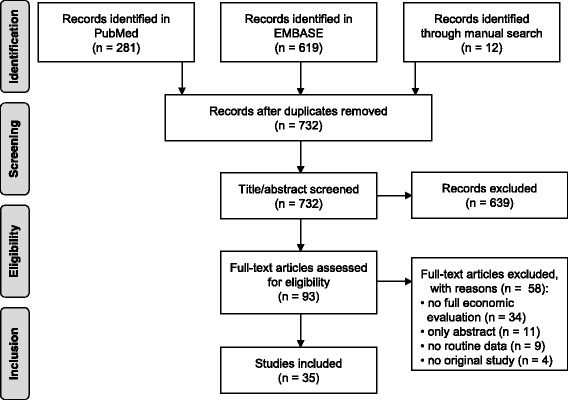
Process of search and study selection

The search resulted in a total of 912 identified records. Of these, 281 records were identified in PubMed, 619 in EMBASE and an additional 12 publications were identified through the manual search. After removing duplicates, 732 records were included in the review process and examined by the involved researchers. Based on the information provided in title and abstract 639 publications were excluded. Of the 93 articles screened in full text, 58 records were excluded due to not meeting at least one of the eligibility criteria. The most common reason for exclusion was a study not being a full economic evaluation (*n* = 34). Other identified records were only abstracts (*n* = 11), did not use routine data as a data source (*n* = 9) or were not original studies (*n* = 4). Figure 1 shows a schematic diagram of the search and selection process which was adapted from the PRISMA statement for systematic reviews [11]. A total number of 35 studies was included in this review [2, 3, 18–50].

### Study characteristics

#### General information

**Fig. 2:**
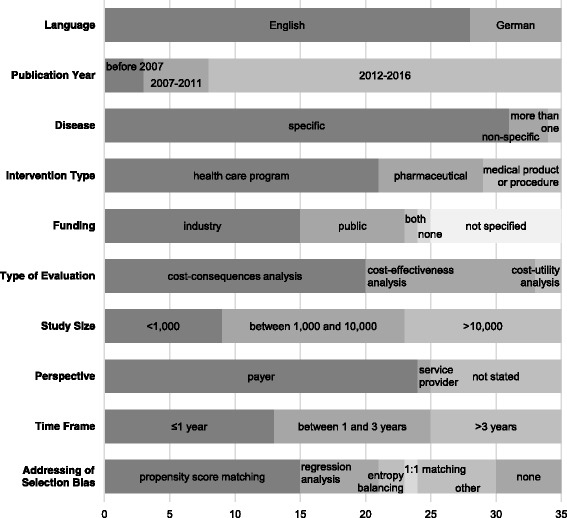
Main characteristics of identified studies

An overview on the main characteristics of the identified studies is shown in Fig. 2. Of the 35 included studies, the majority (*n* = 28, 80.0%) was in English while the language of the remaining studies was German. The publication year of most studies (*n* = 27, 77.1%) was between 2012 and 2016.

Most records (*n* = 24, 68.6%) were published in journals containing only one of the 35 included studies (see Appendix 2). In consequence, the studies were published in a total number of 28 different journals. Regarding the medical indication targeted by the interventions evaluated 88.6% of the studies (*n* = 31) address a specific disease. Table 1 displays a detailed analysis of the medical indications covered. The most common indications were cardiovascular diseases [2, 18, 22, 34, 37, 43, 50] and type 2 diabetes mellitus [25–27, 39, 44, 49]. Figure 2 shows that 21 studies (60.0%) evaluated health care programs such as disease management programs which were assessed in 8 studies (22.9%) alone [21, 25–27, 37, 39, 43, 49]. Evaluations were also performed on pharmaceuticals in 8 studies (22.8%) [30, 35, 41, 44–48] and medical products or procedures in 6 studies (17.1%) [2, 3, 23, 24, 36, 50]. Funding can mainly be ascribed to industry (*n* = 15, 42.9%) – generally the involved health insurance fund or product manufacturer – or public sources (*n* = 8, 22.9%). Public sources mainly include public foundations such as the German Academic Research Foundation or ministries like the German Federal Ministry of Education and Research. One study (2.9%) had both industry and public funding, 1 study (2.9%) was not funded, and 10 studies (28.6%) did not specify their source of funding (Fig. 2).

**Table 1:** Medical indications addressed

Medical indication	Number	Percent
Cardiovascular diseases	7	20.0%
Diabetes mellitus type 2	6	17.1%
Schizophrenia	4	11.4%
Indications related to osteoporosis	3	8.6%
Chronic obstructive pulmonary disease	2	5.7%
Menorrhagia	2	5.7%
Alcohol-dependency	1	2.9%
Cancer	1	2.9%
Femur fractures	1	2.9%
NIndications for penetrating keratoplasty	1	2.9%
Neuropathic pruritus	1	2.9%
Rotavirus	1	2.9%
Trauma	1	2.9%
More than one medical indication	1	2.9%
Non-specific	3	8.6%
Total	35	100.0%

#### Study design

With regard to the study design of the identified economic evaluations, no study used monetary values to measure effects and can therefore be classified as a cost-benefit analysis. Two studies (5.7%) measured health benefits in quality-adjusted life years (QALY) as well as life years gained and are categorized here as cost-utility analysis [41, 44]. A total of 13 studies (37.1%) are cost-effectiveness analysis as they measured consequences in natural units such as life years gained [16]. The remaining 20 studies (57.1%) are classified as cost-consequences analysis as they did not isolate a single consequence or aggregate consequences into a single measure [16]. Thirteen studies (37.1%) reported the type of economic evaluation by use of the keyword cost-effectiveness. However, five studies (14.3%) used a different classification than that of this review as they include the two cost-utility analyses as well as three cost-consequences analyses. Most economic evaluations (*n* = 24, 68.6%) were conducted from a payer’s perspective. In most cases this was the statutory health insurance. One analysis (2.9%) took the perspective of the service provider and ten studies (28.6%) did not explicitly state the perspective of their analysis. In 25 studies (71.4%) the time frame of the analysis was either a maximum of 1 year (*n* = 13, 37.1%) or between 1 and 3 years (*n* = 12, 34.3%). The remaining ten studies (28.6%) had time frames of more than 3 years and up to a lifetime horizon.

A summary of the main outcomes and costs regarded in the economic evaluations can be found in Table 2. The majority of studies reported effect parameters either connected to resource use such as number of hospitalizations and length of stay (*n* = 22, 62.9%) or mortality including among others life years and survival rate (*n* = 17, 48.6%). The most common cost category evaluated in almost every study was inpatient treatment (*n* = 32, 91.4%). While all 35 studies reported costs and effects separately, 23 studies (68.6%) did not include a summary measure such as the incremental cost-effectiveness ratio (ICER) or net-benefit. Eleven studies (31.4%) additionally reported ICERs and 1 study (2.9%) reported values for both ICER and net benefit.

**Table 2:** Main outcome and cost categories included in the economic evaluations analyzed

Outcome category	Number	Percent	Cost category	Number	Percent
Connected to resource use	22	62.9%	Inpatient treatment	32	91.4%
Mortality-related	17	48.6%	Medication	25	71.4%
Complications	7	20.0%	Outpatient treatment	15	42.9%
Incidence of original disease	7	20.0%	Intervention	10	28.6%
Incidence of comorbidities	4	11.4%	Medical aids/remedies	8	22.9%
Patient-reported	3	8.6%	Rehabilitation	6	17.1%
QALY	2	5.7%	Sickness benefits	6	17.1%
Other	10	34.3%	Other/not specified	6	17.1%

**Fig. 3:**
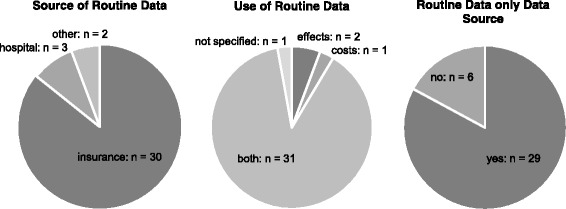
Source and use of routine data in analyzed studies

An overview of the usage of routine data is displayed in Fig. 3. In short, it shows that most studies are based on health insurance data, used routine data to determine both costs and effects and had no other data source. Of the 30 studies (85.7%) involving insurance data, 12 studies (34.3%) are based solely on data from at least one AOK (“Allgemeine Ortskrankenkasse”) [3, 18–20, 28, 29, 32, 33, 35, 38, 40, 43]. Six studies (17.1%) obtained data from today’s “Barmer Ersatzkasse” [21, 25–27, 31, 49], 5 studies (14.3%) involved only the TK (“Techniker Krankenkasse”) [2, 30, 39, 42, 47], and 1 (2.9%) study used data from the SBK (“Siemens-Betriebskrankenkasse”) [22]. All of these are SHI funds. Of the remaining studies, 4 (11.4%) used claims data from several different health insurance funds – 2 of which (5.7%) specified which funds were involved [45, 46]. The other two studies (5.7%) referred to a SHI database [24, 36]. Finally, two studies (5.7%) did not specify which individual fund supplied the data [34, 37]. Of the five studies (14.3%) not based on insurance data, three studies (8.6%) obtained hospital data [23, 48, 50], one study (2.9%) used the German Trauma Registry [41], and one study (2.9%) referenced the International Marketing Services Health (IMS) database as a source [44]. Detailed information on study characteristics can be found in Additional file 1.

#### Analytical approach

As most analyses are based on routine data as a sole data source, a majority of 30 studies (85.7%) considered selection bias at least in part. Five studies (14.3%) did not address the topic of selection bias. While methods for handling selection bias vary, the most common approach was propensity score matching – which was used in 42.9% of the studies (*n* = 15) – followed by different kinds of regression analyses used in 17.1% of the studies (*n* = 6). The remaining 25.7% (*n* = 9) applied other methods including entropy balancing and difference-in-differences [20, 21] and 1:1 matching [32] (see Fig. 2). Data linkage of divergent data sources was rarely applicable and not addressed in any of the studies. This can be ascribed to the fact that most studies had no other data source than routine data. Where data linkage was considered, it referred to linking inpatient and outpatient data. Two studies (5.7%) [33, 38] mentioned the use of identification numbers whereas one study (2.9%) [35] separated the analysis of inpatient and outpatient cases as linkage of treatments was not possible. Regarding the software programs used for the statistical analysis performed, several studies used more than one software. The most commonly applied software was SAS (*n* = 21, 60.0%). Other programs included SPSS (*n* = 5, 14.3%), MS SQL (n = 5, 14.3%), R (*n* = 4, 11.4%), and MS Excel (n = 2, 5.7%). Four studies (11.4%) (additionally) used other software and a total of 7 studies (20.0%) did not state the software used for analysis.

Propensity score matching (PSM) as the most common method for dealing with selection bias was analyzed in detail. The propensity score predicts the probability of an individual being assigned to the treatment group given a function of observed covariates [51]. Generally, the score is estimated by logistic regression – as was the case in 86.6% (*n* = 13) of the 15 studies that applied PSM [2, 3, 22–27, 31, 34, 42, 43, 49]. Two of these studies (13.3%) gave no details on the estimation method [36, 39]. Two studies (13.3%) combined PSM with exact matching of individuals in one or more characteristic (e.g. age). In 1 study (6.6%), both regression analysis and PSM were conducted. Individuals of the treatment and control group were mostly matched 1:1 either with replacement (*n* = 2, 13.3%) – meaning one individual can be matched to more than one other – or without replacement (*n* = 8, 53.3%). Two economic evaluations (13.3%) gave no details on the 1:1 matching algorithm. The remaining 3 studies (20.0%) applied other or unspecified matching methods. In most studies (*n* = 10, 66.6%) the performance of PSM (goodness of fit) was analyzed using standardized differences to compare the propensity score matched treatment and control groups. One of these studies (6.6%) additionally compared the propensity score distributions after matching. Four studies (26.6%) conducted a baseline comparison of the groups after matching while 1 study (6.6%) described no comparison of the baseline groups.

### Reporting quality

**Fig. 4:**
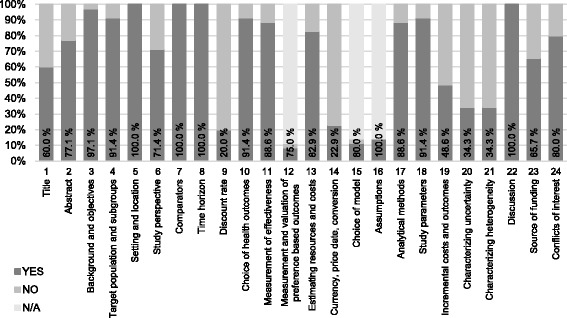
Reporting quality assessment according to CHEERS presented as percentages of studies meeting each checklist item

In total, 15 studies (42.9%) satisfied at least 80% of the applicable items of the CHEERS checklist and could therefore be classified into the group with higher reporting quality. The remaining 20 studies (57.1%) met less than 80% of the CHEERS criteria. All studies included in this review met more than 42% of the criteria analyzed. As three of the 24 checklist items were only evaluated if they were applicable, the minimum absolute requirement varied from study to study. Overall, the studies had to satisfy a minimum number of 17 criteria to meet the benchmark. The three items only evaluated when applicable – items 12, 15 and 16 of the checklist – were rarely assessed. This was due to the fact that few studies included preference based outcomes making their measurement and valuation irrelevant (item no. 12). The items on choice of model (no. 15) and model assumptions (no. 16) were also disregarded in the majority of the studies as most economic evaluations were not model-based. Figure 4 summarizes the assessment of every CHEERS item across all analyzed studies. There were a number of items in which studies frequently lacked adequate reporting according to CHEERS. These criteria included those addressing the reporting of discount rates (item no. 9), currency, price date, and conversion (no. 14) as well as the items on characterizing uncertainty (no. 20) and heterogeneity (no. 21). Each of these items was met by 12 studies at most.

## Discussion

On the basis of 35 systematically identified studies, this review provides an overview of the state and quality of recent health economic evaluations applying routine data in Germany. The number of full economic evaluations using routine data published in the past 5 years has significantly increased compared to the years before 2012. In fact, over three quarters of the included studies are dated after 2011 – without applying an overall limitation to the year of publication during the literature search. Despite the small sample evaluated in this analysis, it displays a clear development towards routine data becoming a more common source for health economic evaluations in Germany. This result is consistent with the findings of Schreyögg and Stargardt from 2012 [9] and a recent review by Kreis et al. on German claims data analyses in general [1].

While the term routine data not only refers to health insurance claims data, this type of routine data was by far the most common in the identified evaluations. One of the reasons for the increasing application of predominantly claims data is data accessibility. In most cases, as shown by Kreis et al. [1], claims data was obtained from an individual SHI fund. Accessibility to routine data sources such as the DIMDI database, however, continues to be limited. This is clearly demonstrated by the small number of publications based on this database up to date [5]. Whether or not the SHI database named as a data source by two of the studies evaluated in this review refers to the DIMDI database could not be assessed.

Aside from improvements in data access, increased application of routine data can be connected to the types of interventions evaluated. Over half of this review’s studies was performed to assess the costs and effects of health care programs such as disease management programs (DMPs). German DMPs are of particular interest in this context as *(1)* their evaluation is mandatory in certain aspects, *(2)* conventional study designs such as randomized controlled trials are generally not feasible for their evaluation and *(3)* SHIs have a large financial interest in these programs. Especially the third factor is enhanced by the rising numbers of DMPs and their enrollees [52]. Together these drivers contribute to more insurances performing economic evaluations based on claims data and can explain the high proportion of this kind of analysis presented here.

The dominance of health care programs is closely connected to the medical indications addressed by the included studies. This becomes clear in the case of type 2 diabetes mellitus – the second most common indication in this review – where 5 out of 6 studies evaluated DMPs. The frequency of studies on both type 2 diabetes mellitus and cardiovascular diseases can be ascribed to the high prevalence and associated costs of these indications [53]. Therefore, SHI funds in particular are interested in the investigation of treatment and prevention programs for these diseases. As such, this finding indicates that the purpose of most included studies was to support SHI decisions. This conclusion is supported by the perspective and cost categories chosen. In most cases, the considered costs included only those relevant to insurance funds such as direct medical costs.

Apart from identifying an increase in publications and a focus on insurance claims data, other main findings of this review refer to the application of routine data in the economic evaluations performed. In short, routine data was primarily used to evaluate costs as well as effects of an intervention. Applying routine data for both components of health economic evaluations is noteworthy due to the challenges related to measuring effects in routine data analysis [14]. Additionally, German insurance claims data is confined with regard to content that can be used to measure effects. Hence, there have been methodological publications on routine data analyses that suggest limited applicability and potential of claims data for health economic evaluations [4]. Nevertheless, the fact that most studies evaluated in this review apply routine data equally to costs and effects corresponds to the results of Schreyögg and Stargardt from 2012 [9]. In their overview, the authors identified studies without data linkage as the greater part of economic evaluations based on insurance claims data.

The evaluation of analytical approaches regarding routine data application demonstrated that the consideration of routine data specific features varied in the included studies. While the majority of studies took selection bias into account, data linkage and uncertainty analysis were less present. With respect to selection bias, both the methods and the quality of consideration showed a wide variety. The need for improvement is demonstrated in missing details on the adjustments performed and missing discussion of the applicability or performance of the chosen methods. Insufficiencies in these areas substantially reduce the reliability of the study results reported. Whereas data linkage was rarely applied, the RECORD statement requires an explanation on whether or not data linkage was included. This was omitted in the majority of studies. Overall, the methodological specifics of routine data were incorporated into the study design of most evaluations at least in part.

With regard to the type of economic evaluation performed, most interventions were analyzed within the framework of cost-consequences or cost-effectiveness analyses. In due consideration of the study perspective, the analysis of cost categories revealed that many evaluations did not include all relevant costs. As the valuation of health outcomes in QALY and monetary units has been the topic of an ongoing debate in Germany, it is not surprising that only two cost-utility analyses and no cost-benefit analysis were identified in this review. The fact that information on QALY is not routinely collected in administrative data additionally contributes to the small number of cost-utility studies conducted. While most economic evaluations regarded in this review were classified as cost-consequences analyses, little more than one third of the studies explicitly stated the type of economic evaluation. Furthermore, for the purpose of this review an explicit classification was defined as the mere mentioning of the term cost-effectiveness e.g. as a keyword.

This insight is linked to another study characteristic assessed in this review: the quality of reporting and associated methodological deficits. With regard to the reporting quality of the included studies, the CHEERS based analysis revealed that more than half of the studies did not reach the benchmark of meeting 80% of the checklist’s criteria. This is a clear indicator that a minimum of reporting standards for economic evaluations was frequently not met. For the most part, the lack of transparency concerned methodological aspects of economic evaluations. While in some cases the absence of addressing an item can be put into perspective by considering the study design – such as ascribing missing discount rates to time horizons of under 1 year – other items show severe reporting deficits. This is exemplified in the insufficient reporting of incremental costs and outcomes in cost-consequences analyses or of ICERs in cost-effectiveness analyses present in over half of the studies. Another indication of questionable overall reporting quality according to CHEERS is the inadequate addressment of uncertainty and heterogeneity in almost two thirds of the studies.

This review as well as its results are subject to several limitations regarding both the identification of studies and the review process. First, the number of databases and resulting studies was restricted. Despite the fact that the systematic literature search in the databases PubMed and EMBASE was complemented by an extensive manual search, it is possible that studies fitting the inclusion criteria were omitted. Second, authors refer to routine data using various terms and only a restricted set of synonyms was used within the systematic search. However, these terms are comparable to those of related reviews [1, 7] and it can be assumed that most relevant publications used the included expressions. An additional limitation of the search strategy is restricting the search component on routine data and economic evaluations to the search fields title, abstract and keywords. In consequence, a study in which the routine data source was not a focus may have been omitted by the search algorithm.

Regarding limitations of the review process, the appraisal of the studies’ quality was mainly restricted to their quality of reporting. In this context, it is important to note that although the routine data specific RECORD statement [15] was considered within the analysis of analytical approaches, the structured evaluation of reporting quality was based only on the CHEERS checklist. This decision was made to avoid a repetitive analysis with two checklists and enable a transparent evaluation based on an established instrument. As the focus of this review was on the quality of economic evaluations, the CHEERS checklist was chosen as the most suitable instrument. While this allowed an adequate assessment of most study features, the review process revealed a need for a routine data specific modification of the CHEERS checklist. This refers to both adding certain items such as reporting of bias and making adjustments to individual items such as estimating resource use before costs.

A concluding limitation is connected to the classification of the studies analyzed in this review. While the authors of several publications did not explicitly classify their study as an economic evaluation, they were defined and evaluated as such for the purpose of this review. This circumstance is important to consider especially with regard to the reporting quality. While some items of the CHEERS checklist are relevant for any kind of study, it is likely that some of the mentioned specifics were not addressed by the studies’ authors since it was not their intention to perform an economic evaluation. Accordingly, the relatively low percentage of CHEERS criteria fulfilled has to be seen in this context.

Notwithstanding the mentioned limitations, this review offers a comprehensive overview of the current state of full economic evaluations based on routine data in Germany. On the one hand, it acknowledges the potential of routine data by demonstrating its increased application to measure both costs and effects of health care interventions. In addition to this, it shows that most evaluations were performed to support SHI decisions on health care programs. On the other hand, this review reveals that while most studies address the particularities of routine data analyses like selection bias, methods and quality of consideration differ. Individual studies show a clear lack of transparency with regard to data linkage, adjustment for selection bias or details on matching methods. Moreover, this review reveals deficiencies in reporting quality as many studies do not meet a minimum requirement of reporting standards for economic evaluations. Taken together, these quality issues demonstrate the caution both researchers and practitioners should have when interpreting economic evaluations based on routine data. Methodological deficits in particular illustrate the need for structured consideration of appropriate guidelines when conducting and reporting routine data analyses. Additional implications for further research entail the development of adjusted reporting guidelines for economic evaluations based on routine data. Furthermore, this review can be used as a point of reference for further research such as international comparisons of the use of routine data for economic evaluations.

## Conclusions

The main objective of this review was to assess the characteristics and reporting quality of economic evaluations based on German routine data. Of 35 identified studies, most were classified as cost-consequences analyses using health insurance claims data to evaluate both costs and effects – mainly of health care programs. While most studies addressed the particularities of routine data as a data source, many considerations were not exhaustive. With respect to the reporting quality of the studies, less than half fulfilled a benchmark of meeting at least 80% of the applicable CHEERS criteria. This indicates potential for improvement in the reporting of economic evaluations based on routine data. Implications for future research include the careful consideration of the methodological specifics connected to health economic evaluations based on routine data. As the reliability of studies strongly depends on a sound analytical approach, cautious interpretation of results is required for researchers and practitioners. A better understanding of economic evaluations based on routine data as well as more transparent, high quality research could be promoted by specialized reporting guidelines.

### Additional file

Additional file 1: Study characteristics presented for each included study according to structured template. Includes detailed information on study characteristics as analyzed for the purpose of this review. (PDF 1440 kb)

## Appendix 1

**Table 3:** Search strategies in PubMed and EMBASE

PubMed search	EMBASE search
claims data[Text WOrd] OR routine data[Text Word] OR insurance data[Text Word] OR administrative data[Text Word] OR administration data[Text Word] OR sickness funds data[Text Word] OR secondary data[Text Word]	(claims data OR routine data OR insurance data OR administrative data OR administration data OR sickness funds data OR secondary data).ti,ab,kw.
AND	AND
“Germany”[MeSH] OR German OR Germany	exp Germany/OR German OR Germany
AND	AND
“costs and cost analysis”[MeSH] OR cost[Text Word] OR costs[Text Word]	exp Health Economics/OR (cost or costs).ti,ab,kw.

## Appendix 2

**Table 4:** Journals of publication of included studies

Journal	Number	Percent
Eur J Health Econ	3	8.6%
Health Policy	3	8.6%
Z Evid Fortbild Qual Gesundhwes	3	8.6%
Dtsch Arztebl Int	2	5.7%
Others^a^	24	68.6%
Total	35	100.0%

^a^Journals with one included study each

## Abbreviations

CHEERS: Consolidated Health Economic Evaluation Reporting Standards
DIMDI: German Institute of Medical Documentation and Information
DMPs: Disease management programs
ICER: Incremental cost-effectiveness ratio
IQWiG: Institute for Quality and Efficiency in Health Care
PRISMA: Preferred Reporting Items for Systematic Reviews and Meta-Analyses
PSM: Propensity score matching
QALY: Quality-adjusted life years
RECORD: REporting of studies Conducted using Observational Routinely-collected health Data
SHI: Statutory health insurance
