# Supplementary material for: Health economic evaluations based on routine data in Germany: a systematic review
Source: BMC Health Serv Res. 2018 Apr 10;18:268. doi: 10.1186/s12913-018-3080-3 (PMC5894241; doi:10.1186/s12913-018-3080-3)
Supplement: Supplementary file 1 — Study characteristics presented for each included study according to structured template. Includes detailed information on study characteristics as analyzed for the purpose of this review. (PDF 1440 kb) [file 12913_2018_3080_MOESM1_ESM.pdf]

Additional file 1: Study characteristics presented for each included study according to structured template

Study	Journal	Language	Disease	Intervention	Funding	Evaluation Type	Type stated	Routine Data (RD) source	Use of RD	Only RD?	Study Size	Perspective	Time Frame of analysis
**Abbas et al. 2013 [18]**	Rehabilitation (Stuttg)	GER	**SPECIFIC** cerebral infarction/ stroke; ICD-10 163, 164; after discharge; geriatric patients	**HCP** geriatric departments in hospitals (§109 SGB V) **VS** geriatric rehabilitation facilities (§111 SGB V)	**PUBLIC** Hessian Ministry of Social Affairs	CCA	no	**Insurance** AOK WIdO AOK Bayern, Hessen Niedersachsen, Rheinland Pfalz, Saarland, Sachsen, Thüringen	costs and effects	yes	n = 3472	**payer** statutory nursing care and health insurance	1 year
**Abbas et al. 2015 [19]**	Z Gerontol Geriatr	GER	**SPECIFIC** femur fractures; ICD-10: S72; after discharge; geriatric patients	**HCP** geriatric departments in hospitals (§109 SGB V) **VS** out-of-hospital rehabilitation facilities (§111 SGB V)	**PUBLIC** Hessian Ministry of Social Affairs	CCA	no	**Insurance** AOK WIdO AOK Bayern, Hessen Niedersachsen, Rheinland Pfalz, Saarland, Sachsen, Thüringen	costs and effects	yes	n = 6089	**not stated**	1 year
**Achelrod et al. 2016 [20]**	Eur J Health Econ	ENG	**1 SPECIFIC** COPD patients; ICD-GM-10 (J44)	**HCP** telemonitoring for COPD patients **VS** no telemonitoring	**INDUSTRY** AOK Bayern and SHL Telemedizin	CEA	yes	**Insurance** AOK Bayern	costs and effects	yes	n = 7698	**payer** statutory health insurance	1 year
**Achelrod et al. 2016 [21]**	Health Policy	ENG	**SPECIFIC** chronic obstructive pulmonary disease (COPD) patients; ICD-GM-10 (J44)	**HCP** disease management program (DMP) for COPD patients **VS** no participation in any DMP for COPD	**PUBLIC** German Academic Research Foundation (DFG)	CEA	yes	**Insurance** Barmer GEK	costs and effects	yes	n = 215104	**payer** statutory health insurance	3 years

Outcomes	Costs	Summary Measure unit	Sel. Bias considered	Methods for Sel. Bias	Details on Methods	Data linkage	Handling of Uncertainty	Software
mortality; rehospitalization due to ischemic stroke; rehospitalization due to fracture	costs for inpatient and outpatient care with and without long-term care	**Seperate** differences in mortality and rehospitalization as hazard ratio (HR); costs in euros	yes	regression analysis	quantile regression for excess cost analysis, treatment and further co-variables as variables; cox regression to measure influence of treatment on time to death and rehospitalization, various confounders	not applicable for external sources; not addressed for linkage of inpatient and outpatient data	confidence intervals	**SAS 9.2; MS-SQL-Server 2008**
mortality; rehospitalization due to femoral fracture	costs for inpatient and outpatient care with and without long-term care	**Seperate** differences in mortality and rehospitalization as HR; costs in euros	yes	regression analysis	quantile regression for excess cost analysis, treatment and further co-variables as variables; multivariate cox regression to measure influence of treatment on time to death and rehospitalization, various confounders	not applicable for external sources; not addressed for linkage of inpatient and outpatient data	confidence intervals	**SAS 9.2**
mortality; years of life lost; number of hospitalizations; number of outpatient physician visits; average length of stay; proportion of hospitalized patients; number of pharmaceutical prescriptions	direct medical costs for inpatient and outpatient treatment, pharmaceuticals, and rehabilitation	**ICER and seperate** ICER not applicable, mortality HR; cost savings in euros	yes	entropy balancing and difference-in-difference	(1) reweighting algorithm to remove imbalances in the mean and variance of pre-specified, observed covariates (e.g. age, sex) (2) DiD estimation to determine differences in outcomes due to unobserved factors, comparing differences after and before intervention	not applicable for external sources; not addressed for linkage of inpatient and outpatient data	change in response to (1) exclusion of deceased individuals, (2) truncation of high-cost cases and (3) intention-to-treat analysis	not stated
mortality; morbidity; number of inpations days/stay; average length of stay; proportion of hospitalized patients; number and proportion of outpatient physician visits; number of prescriptions and exacerbations	direct medical costs for inpatient and outpatient treatment, pharmaceuticals, rehabilitation and devices/medical appliances; administrative expenses for the DMP	**ICER and seperate** euros per life year gained; mortality rate; mortality hazards ratio; indicator values of morbiditay costs in euros	yes	entropy balancing and difference-in-difference	(1) reweighting algorithm to remove imbalances in the mean and variance of pre-specified, observed covariates (e.g. age, sex) (2) DiD estimation to determine differences in outcomes due to unobserved factors, comparing differences after and before intervention	not applicable for external sources; not addressed for linkage of inpatient and outpatient data	impact of (1) mortality, (2) misdiagnosis of COPD, (3) exposure time to DMP and (4) drop-out from DMP	not stated

Study	Journal	Language	Disease	Intervention	Funding	Evaluation Type	Type stated	Routine Data (RD) source	Use of RD	Only RD?	Study Size	Perspective	Time Frame of analysis
**Aljutaili et al. 2014 [22]**	BMC Health Serv Res	ENG	**SPECIFIC** CHD (coronary heart disease) prevention	**HCP** KardioPro (individualised prevention program to prevent CHD) **VS** non-participants	**PUBLIC AND INDUSTRY** SBK and Helmholtz Zentrum München	CEA	yes	**Insurance** SBK (Siemens-Betriebskrankenkasse)	costs and effects	yes	n = 26224	**payer** statutory health insurance	mean observation period 2.4 years
**Bäumler et al. 2012 [2]**	Appl Health Econ Health Policy	ENG	**SPECIFIC** patients with acute myocardial infarction (AMI); ICD 10-I21	**MP** drug eluting stents (DES) **VS** bare-metal stents (BMS)	**PUBLIC** Technical University of Berlin	CEA	yes	**Insurance** TK (Techniker Krankenkasse)	costs and effects	yes	n = 1438	**payer** statutory health insurance	1 year
**Bäumler et al. 2014 [23]**	Int J Technol Assess Health Care	ENG	**SPECIFIC** patients who underwent a penetrating keraplasty	**MP** human leukocyte antigen matching **VS** no matching	**PUBLIC** German Academic Research Foundation (DFG)	CEA	yes	**Hospital** Freiburg University Eye Hospital	effects	no	n = 721	**payer** statutory health insurance	6 years and 2 months
**Bischoff-Everding et al. 2016 [24]**	Int J Womens Health	ENG	**SPECIFIC** women with monorrhagia; ICD-10-GM-N92.0, N92.1 and N92.3-6	**MP** endometrial radiofrequency ablation **VS** other ablation techniques	**INDUSTRY** Hologic Deutschland GmbH	CCA	no	**Insurance** SHI database (not specified)	costs and effects	yes	n = 88	**payer** statutory health insurance	2 years and 3 months
**Drabik et al. 2012 [25]**	Diabetes Res Clin Pract	ENG	**SPECIFIC** type 2 diabetes mellitus (T2DM)	**HCP** DMP **VS** routine care	**NONE**	CEA	yes	**Insurance** Barmer GEK	costs and effects	yes	n = 39776	**payer** statutory health insurance	19 years

Outcomes	Costs	Summary Measure unit	Sel. Bias considered	Methods for Sel. Bias	Details on Methods	Data linkage	Handling of Uncertainty	Software
event-free time in days (myocardial infarction, stroke, death); death-free time in days	hospital costs; pharmaceutical costs; physician costs; other costs (e.g. physiotherapy, laboratory resources, services like acupuncture and sickness benefits)	**ICER and seperate** euros per event free year in different subgroups; event free time in days; costs in euros	yes	propensity score matching	propensity score (PS) computed by logistic regression; stepwise variable selection; approximate nearest neighbour 1:1 maching without replacement; baseline comparison after matching	not applicable for external sources; not addressed for linkage of inpatient and outpatient data	deterministic approach with alternate discount rates bootstrapping with 10.000 on cost and effect samples to calculate ICER	**SAS 9.2**
mortality (365-day survival)	inpatient costs (mean cost of treatment, incremental cost 30 days after implantation)	**ICER and seperate** euros per life saved; average costs in euros; difference in 365-day survival in percent	yes	propensity score matching	PS computed by logistic regression; theoretical considerations and statistical methods (fast false selection rate) for variable selection; 1:1 optimal matching with replacement; calliper of 0.0005; goodness of fit by standardized differences (SDF)	not applicable for external sources; not addressed for linkage of inpatient and outpatient data	bootstrapping for ICER, restricted definition of costs, re-estimating model, stratified analysis	**SAS 9.2**
graft survival	direct costs (costs of graft, cost of hospital admission, cost of keratoplasty procedure, cost of follow-up visists, medication during follow-up)	**ICER and seperate** euros per additional day of graft survival; rejection-free graft survival time in days; incremental costs in euros	yes	propensity score matching	PS computed by logistic regression; a priori selection of variables; 1:1 nearest neighbor matching with replacement; calliper of 0.01; SDF to evaluate goodness-of-fit (>10%)	not applicable	multivariate sensitivity analysis regarding (1) extrapolation of survival and (2) cost of intervention	**SAS**
rate of relapse (rate of repeat diagnoses of menorrhagia after index treatment); number of uterine treatments for recurrent menorrhagia	average costs of inpatient care; outpatient care; pharmaceuticals (outpatient setting); sick pay; remedies; medical aids; total costs	**Seperate** percentage of repeat diagnoses and frequency of hysterectomy; additional costs in euros	yes	propensity score matching	PS computed by logistic regression; a priori selection of variables; GenMath matching algorithm with a caliper of 0.5; SDF and check of distributions after matching	not applicable for external sources; not addressed for linkage of inpatient and outpatient data	no	**SAS 9.2;** Microsoft Office **Excel 2010**; **R** for GenMatch algorithm
life years gained; number of inpatient stays; duration of inpatient stays	average basic total costs (drug costs and hospital costs)	**ICER and seperate** euros per life-year gained; life years; costs in euros	yes	propensity score matching	PS computed by logistic regression; stepwise variable selection; 1:1 matching without replacement with Parson's 5 to 1 method; SDF to evaluate goodness-of-fit (<10%)	not applicable for external sources; not addressed for linkage of inpatient and outpatient data	sensitivity analysis with discount rates of 0 and 7 %	**SAS 9.2; R 2.10.1**

Study	Journal	Language	Disease	Intervention	Funding	Evaluation Type	Type stated	Routine Data (RD) source	Use of RD	Only RD?	Study Size	Perspective	Time Frame of analysis
**Drabik et al. 2012 [26]**	Popul Health Manag	ENG	**SPECIFIC** type 2 diabetes mellitus (T2DM)	**HCP** DMP **VS** routine care	**NOT SPECIFIED**	CEA	no	**Insurance** Barmer GEK	costs and effects	yes	n = 39776	**not stated**	3 years
**Drabik et al. 2012 [27]**	Z Evid Fortbild Qual Gesundhwes	GER	**SPECIFIC** type 2 diabetes mellitus (T2DM)	**HCP** DMP **VS** routine care	**NOT SPECIFIED**	CCA	no	**Insurance** Barmer GEK	costs and effects	yes	n = 39776	**not stated**	3 years
**Driessen et al. 1999 [28]**	Nervenarzt	GER	**SPECIFIC** alcohol-dependent patients	**HCP** extended alcohol withdrawal treatment program (II) **VS** medical detoxification program (I)	**NOT SPECIFIED**	CCA	yes	**Insurance** AOK Lübeck	costs and effects	yes	n = 94	**not stated**	10 years
**Freund et al. 2016 [29]**	Ann Intern Med	ENG	**MORE THAN ONE MEDICAL INDICATION** type 2 diabetes, COPD, chronic heart failure, high likelihood of hospitalization	**HCP** protocol-based care management by medical assistants **VS** routine care	**INDUSTRY** AOK Baden-Wuerttemberg and AOK Bundesverband	CCA	no	**Insurance** AOK Baden-Württemberg	effects	no	n = 2076	**not stated**	1 year
**Frey et al. 2014 [30]**	Eur J Health Econ	ENG	**SPECIFIC** patients hospitalized with schizophrenia; ICD-10-GM F20.x	**Pharmaceutical** long-acting injectable risperidone (LAI-RIS) **VS** long-acting injectable flupentixol (LAI-FLX)	**INDUSTRY** Bayer Vital	CEA	yes	**Insurance** TK (Techniker Krankenkasse)	costs and effects	yes	n = 935	**payer** statutory health insurance	2 years

Outcomes	Costs	Summary Measure unit	Sel. Bias considered	Methods for Sel. Bias	Details on Methods	Data linkage	Handling of Uncertainty	Software
mortality	mean daily costs (drug costs and hospital costs); total costs (including DMP lump sum for administration and medical costs)	**Seperate** mean survival time in days; daily hospital and total costs in euros	yes	propensity score matching	PS computed by logistic regression; stepwise variable selection; 1:1 matching without replacement with Parson's 5 to 1 method; SDF for goodness-of-fit (<10%)	not applicable for external sources; not addressed for linkage of inpatient and outpatient data	no	**SAS 9.2**
mortality; complications; number of inpatient stays; duration of inpatient stays	average basic total costs (drug costs and hospital costs)	**Seperate** mortality in absolute deaths and mortality rate; costs in euros	yes	propensity score matching	PS computed by stepwise logistic regression; matching with Parson's 5 to 1 method; 1:1 nearest neighbor approach without replacement; SDF to evaluate goodness-of-fit (<10%)	not applicable for external sources; not addressed for linkage of inpatient and outpatient data	no	**SAS 9.2**
number of hospitalizations; length of hositalizations; days of incapacity to work; days of financial substitution for incapacity to work	total inpatient cost; treatment cost; therapy cost; inpatient cost	**Seperate** number of hospitalizations in days; length of stay in days; days of financial support; costs in euros	no	none	-	not applicable for external sources; not addressed for linkage of inpatient and outpatient data	no	**SPSS**
number of hospitalizations; mortality; quality of life (patient-reported)	intervention costs	**Seperate** number of hospitalizations and relative risk (RR); HR of mortality; quality-of-life scores; intervention costs in euros	yes	cluster-randomized trial	random allocation of primary care practices; 1:1 block randomization with variable block lengths; stratified randomization according to poppulation density; blinded assessment of end points by statistician	not addressed	sensitivity analysis with multivariate models	**R 3.2.0** for Windows (glmmADMB and coxme package); **Case Smart Suite Germany 0.7**
days in hospital; probability of c-medication; 1-year treatment discontinuation in percent; 2-year treatment discontinuation in percent	inpatient treatment cost; outpatient treatment cost; antipsychotic medication cost; co-medication cost; total cost	**ICER and seperate** incremental costs per hospital day avoided; days in hospital; costs in euros	yes	regression analysis	multivariate regression methods to adjust for patient heterogeneity; cost, hospital days and probability of co-medication as function of effectiveness conditioning on risk-adjusted covariates	not applicable (model)	alternate structural specifications of the model; probabilistic sensitiity analyses (Monte-Carlo, 10.000 iterations); sampling of normal correlated coefficients for each covariate; subgroup analysis	**SAS 9.2;** **R** (stats- and pscl packages) for statistical analysis; **MS Excel** for decision analysis model

Study	Journal	Language	Disease	Intervention	Funding	Evaluation Type	Type stated	Routine Data (RD) source	Use of RD	Only RD?	Study Size	Perspective	Time Frame of analysis
**Gaertner et al. 2013 [31]**	Health Policy	ENG	**SPECIFIC** cancer patients in the last 6 months of life; ICD-10 C00-C76	**HCP** treatment in an inpatients palliative care (PC) unit **VS** no PC	**PUBLIC** Dr. Werner Jackstädt-Foundation	CCA	no	**Insurance** Barmer GEK	costs and effects	yes	n = 1682	**payer** statutory health insurance	6 months
**Goltz et al. 2013 [32]**	Pharmaco epidemiol Drug Saf	ENG	**SPECIFIC** patients with osteoporosis and previous osteoporotic fractures	**HCP** program of integrated care (IC) **VS** insurants not enrolled in program	**INDUSTRY** AOK PLUS	CCA	yes	**Insurance** AOK PLUS	costs and effects	yes	n = 4910	**payer** statutory health insurance	3 years
**Heinrich et al. 2013 [33]**	Osteoporos Int	ENG	**NON-SPECIFIC INTERVENTION**	**HCP** multifactorial fall prevention program **VS** usual care	**PUBLIC** German Federal Ministry of Education and Research	CEA	yes	**Insurance** AOK (Bayern)	costs and effects	yes	n = 33152	**payer** statutory health insurance	1 year
**Hendricks et al. 2014 [34]**	Dtsch Arztebl Int	ENG	**SPECIFIC** patients with chronic heart failure	**HCP** case management program (CMP) **VS** German routine care	**INDUSTRY** participating company health insurers	CCA	no	**Insurance** not specified	costs and effects	yes	n = 1202	**not stated**	4 years and 6 months
**Karmann et al. 2015 [35]**	Eur J Health Econ	ENG	**SPECIFIC** rotavirus cases	**Pharmaceutical** rotavirus (RV) vaccination **VS** no vaccination	**INDUSTRY** GlaxoSmith Kline	CEA	no	**Insurance** AOK PLUS	costs and effects	no	360000 observations	**payer** statutory health insurance	4 years

Outcomes	Costs	Summary Measure unit	Sel. Bias considered	Methods for Sel. Bias	Details on Methods	Data linkage	Handling of Uncertainty	Software
symptom control (opiod prescription); place of death (hospital); aggressiveness of care (prescription of chemotherapy); end-of-life decisions (prescription of artificial nutrition)	mean total health care costs; mean hospital costs; mean medication costs; additional medical services; remedies	**Seperate** percentage of patients receiving opioids and chemotherapy; costs in euros	yes	propensity score matching	PS computed by stepwise logistic regression: 1:1 matching without replacement with Parson's 5 to 1 method; SDF to evaluate goodness-of-fit (<10%)	not applicable for external sources; not addressed for linkage of inpatient and outpatient data	confidence intervals	**SAS 9.2** for statistical analysis and matching
fracture incidence; time to refracture in days; pain (median uptake of analgesics in DDD); medication supply	costs for osteoporosis-related medication; treatment costs for osteoporosis-related fractures; integrated care program costs	**Seperate** percentage of medication per year; fracures; costs in euros	yes	1:1 matching	matching osteoporosis insurants based on age, sex and fracture location (not detailed description)	not applicable for external sources; not addressed for linkage of inpatient and outpatient data	no	**SPSS 17.0** for statistical analysis
mean number of incident femoral fractures; mean days free of femoral fracture; all cause mortality	costs per resident; mean direct costs for femoral fractures; mean total direct costs including intervention costs	**ICER, net benefit and seperate** euros per year free of femoral fracture, adjusted NMB for different WTP for a year free of femoral fracture	yes	regression analysis	multivariate regression model; adjustment for age, gender, level of cara, and size of nursing home; regression analysis to analyze costs and cost-effectiveness using NMB	not applicable for external sources; linkage of different sectors for each individual with identification code	sensitivity analyses performed on intervention costs (decrease and increase by 50%)	**SAS 9.2 PASW Statistics 20** for statistical analysis
hospital admission rate; hospital readmission rate; mortality; number of contacs with physicians per year; hospital stay duration	mean cost per heart failure-related hospital stay; annual heart failure related hospitalization costs	**Seperate** rate of hospital admission/readmission in percent; length of stay in days; costs in euros	yes	propensity score matching	stepwise logistic regression to calculate PS; 1:1 matching; baseline comparison after matching	not applicable for external sources; not addressed for linkage of inpatient and outpatient data	confidence intervals	**SPSS 19** for statistical analysis
number of RV cases; number of RV cases avoided	direct medical costs (inpatient and outpatient treatment); direct non-medical costs (sickness-benefits); costs of vaccination	**ICER and seperate** cost savings per avoided case; number of RV cases; cost savings per 1000 children	no	none	-	analysis of inpatient and outpatients cases seperately (not patients) since linkage of inpatient and outpatient treatment within one quarter not possible	univariate sensitivity analyses varying sensitive parameters (incidence rate, days absence from work) until net cost savings faded out	**MS Excel** for model

Study	Journal	Language	Disease	Intervention	Funding	Evaluation Type	Type stated	Routine Data (RD) source	Use of RD	Only RD?	Study Size	Perspective	Time Frame of analysis
**Kessel et al. 2015 [36]**	Expert Rev Med Devices	ENG	**SPECIFIC** women with monorrhagia; ICD-10-GM-N92.0, N92.1 and N92.3-6	**MP** radiofrequency ablation (RFA) **VS** hysterectomy	**INDUSTRY** Hologic Deutschland GmbH	CCA	no	**Insurance** SHI database (not specified)	costs and effects	yes	n = 214	**payer** statutory health insurance	2 years and 3 months
**Kottmair et al. 2005 [37]**	Health Care Financ Rev	ENG	**SPECIFIC** patients with congestive Heart Failure (CHF)	**HCP** DMP **VS** routine care (RC)	**NOT SPECIFIED**	CCA	no	**Insurance** not specified	costs and effects	yes	n = 185	**payer** health insurance	4 years
**Lange et al. 2014 [3]**	Spine	ENG	**SPECIFIC** patients with osteoporotic vertebral compression fractures (OVCFs); ICD-10 80.-8	**MP** surgical treatment (balloon kyphoplasty (BKP) and percutaneous vertebroplasty (PVP)) **VS** nonsurgical treatment	**INDUSTRY** Medtronic International	CEA	no	**Insurance** AOK Niedersachsen	costs and effects	yes	n = 3607	**payer** statutory health insurance	5 years
**Laux et al. 2013 [38]**	Z Evid Fortbild Qual Gesundhwes	GER	**NON-SPECIFIC INTERVENTION**	**HCP** family-doctor centered care (HzV) enrollment **VS** no enrollment in HzV	**NOT SPECIFIED**	CCA	no	**Insurance** AOK Baden-Wuerttemberg	costs and effects	yes	n = 1443161	**not stated**	1 year

Outcomes	Costs	Summary Measure unit	Sel. Bias considered	Methods for Sel. Bias	Details on Methods	Data linkage	Handling of Uncertainty	Software
type and number of post-surgical morbidities during QoT; complications in delected ICD codes; number of repeated diagnoses and surgical re-treatments	cost of medication; outpatient physician consultations; remedies; medical aids; sick pay hospital and dental consultations, total direct costs	**Seperate** percentage of patients with relevant morbidities needing surgical retreatments; mean number of recodings; costs in euros	yes	propensity score matching combined with exact matching	propensity score matching according to Rosenbaum and Rubin; exact matching by age class; 1:3 nearest neighbor matching with caliper of 0.4; baseline comparison after matching	not applicable for external sources; not addressed for linkage of inpatient and outpatient data	confidence intervals	**SAS 9.2** **MS Excel 2010** for calculations and data preparations
proportion of patients with edemas (patient-reported); quality of care expressed in terms of adherence to therapy guidelines	total annual health care expenditures of insurance company	**Seperate** examples for proportions in percent	in part	pre/post design	-	not applicable for external sources; not addressed for linkage of inpatient and outpatient data	no	not stated
survival rate	total costs; inpatient costs; outpatient costs; pharmacy costs; costs for rehabilitation; sick leave payments; costs for remedies and aids	**Seperate** survival rate in percent; costs in euros	in part	regression analysis and propensity score matching	multivariate cox regression model to evaluate joint effect of covariates on mortality risk to compare operated and nonoperated patients; propensity score matching to compare BKP and PBP group; logistic regression to determine PS; a priori variable selection; 1:1 nearest neighbor matching without replacement; SDF to evaluate goodness-of-fit	not applicable for external sources; merging of data via an identification number	not detailed; change of date of surgery	not stated
number of familiy doctor visits; number of specialist visits; rate of hospitalizations; duration of hospitalizations; rate of re-hospitalizations; rate of polypharmacy; rate of Me-Too pharmaceuticals	costs of pharmacotherapy	**Seperate** numbers of visists; rates in percent; costs in euros	yes	regression analysis	multivariate multilevel regression model; covariates: age, sex, nationality, insurance status, morbidity, duration of enrollment in HzV	not applicable for external sources; merging of data via pseudonymised identification number for insurant and pseudonymised lifelong identification number for doctors	confidence intervals	**SAS 9.2** for statistical analysis **ORACLE MySQL** Community Server 5.5 and **IBM DB2** Workgroup Server 9.7 for data storage and processing

Study	Journal	Language	Disease	Intervention	Funding	Evaluation Type	Type stated	Routine Data (RD) source	Use of RD	Only RD?	Study Size	Perspective	Time Frame of analysis
**Linder et al. 2011 [39]**	Dtsch Arztebl Int	ENG	**SPECIFIC** type 2 diabetes mellitus (T2DM)	**HCP** DMP for T2DM participants **VS** non-participants	**NOT SPECIFIED**	CCA	no	**Insurance** TK (Techniker Krankenkasse)	costs and effects	yes	n = 107590	**not stated**	2 years
**Niedhart et al. 2013 [40]**	Z Orthop Unfall	GER	**SPECIFIC** osteoporosis; ICD-10 M80 or M81	**HCP** Integrated care model osteoporosis **VS** routine care	**NOT SPECIFIED**	CEA	no	**Insurance** AOK Rheinland, Hamburg	costs and effects	yes	n = 22040	**payer** statutory health insurance	3 years
**Rossaint et al. 2007 [41]**	Eur J Trauma Emerg Surg	ENG	**SPECIFIC** severely injured trauma patients	**Pharmaceutical** Recombinant activated factor VII (rFVIIa) **VS** placebo to for control of bleeding in patients with blunt	**INDUSTRY** Novo Nordisk	CUA	yes	**Other** German Trauma Registry	costs and effects	no	n = 143	**payer** statutory health insurance	lifetime
**Schneider et al. 2016 [42]**	Health Policy	ENG	**NON-SPECIFIC INTERVENTION**	**HCP** graded return to work (RTW) program **VS** non-participants	**NOT SPECIFIED**	CCA	no	**Insurance** TK (Techniker Krankenkasse)	costs and effects	yes	n = 11212	**payer** statutory health insurance	1 year and 6 months

Outcomes	Costs	Summary Measure unit	Sel. Bias considered	Methods for Sel. Bias	Details on Methods	Data linkage	Handling of Uncertainty	Software
incidence of specific comorbidities; frequency of emergency inpatient admissions; use of pharmacotherapy; number of outpatient physician contacts; use of outpatient services	hospital costs; drug presciption costs	**Seperate** DALY-weighted incidence of co-morbidities; drug use in DDD; costs in euros	yes	propensity score matching	propensity score interval matching; a priori selection of variables; baseline comparison after matching	not applicable for external sources; not addressed for linkage of inpatient and outpatient data	no	**PASW Statistics 18** and **SAS Enterprise Guide V.4.1** for statistical analysis
hospitalization rate for hip fractures	total costs; total hospital costs; fracture-related hospital costs; inpatient care costs; outpatient care costs; total rehabilitation costs; inpatient rehabilitation; outpatient rehabilitation; remedies and medical aids; total and specific drug costs	**Seperate** hospitalisation rate per 1000 patient years; medication costs in euros	no	none	-	not applicable for external sources; not addressed for linkage of inpatient and outpatient data	no	**SAS** for statistical analysis
survival time in days; QALY	drug aquisitaion costs; total hospital costs; total lifetime costs	**ICER and seperate** euros per life-year gained; euros per QALY gained; cost-effectiveness acceptability curve	no	none	-	not applicable (model)	univariate sensitivity analysis to assess impact of key model parameters: mortality risk, discount rate for costs and effects, long-term costs, residual life expectancy, utility values in remaining life years	not stated
days sick; days with sickness benefits; sickness benefits; sickness benefits per day	outpatient costs; hospital costs; pharmaceuticals; other costs; total costs; total costs without outpatient	**Seperate** sick days; sickness benefits; expenditures	yes	propensity score matching	PS computed by logistic regression; stepwise variable selection; 1:1 matching without replacement (using 4-2 digit greedy matching); SDF to evaluate goodness-of-fit	not applicable for external sources; not addressed for linkage of inpatient and outpatient data	no	not stated

Study	Journal	Language	Disease	Intervention	Funding	Evaluation Type	Type stated	Routine Data (RD) source	Use of RD	Only RD?	Study Size	Perspective	Time Frame of analysis
**Schulte et al. 2016 [43]**	Z Evid Fortbild Qual Gesundhwes	GER	**SPECIFIC** coronary heart disease	**HCP** DMP **VS** routine care	**NOT SPECIFIED**	CCA	no	**Insurance** AOK Baden-Wuerttemberg	costs and effects	yes	n = 382	**not stated**	3 years
**Shearer et al. 2006 [44]**	Pharmacoeconomics	ENG	**SPECIFIC** type 2 diabetes mellitus (T2DM)	**Pharmaceutical** rosiglitazone in combination with other oral agents (two different strategies) **VS** conventional care (three strategies)	**INDUSTRY** GlaxoSmith Kline	CUA	yes	**Other** IMS (International Marketing Services Health) Disease analyser	costs	no	n = 1000	**payer** statutory health insurance	lifetime
**Stargardt et al. 2012 [45]**	J Clin Psychopharmacol	ENG	**SPECIFIC** schizophrenia; ICD-10 F20.0 to F20.9	**Pharmaceutical** atypical **VS** typical antipsychotic treatment for schizophrenia	**INDUSTRY** Janssen Cilag	CCA	no	**Insurance** TK (Techniker Krankenkasse), AOK Baden-Wuerttemberg, AOK Westfalen-Lippe, AOK Berlin	costs and effects	yes	n = 8610	**payer** statutory health insurance	1 year
**Stargardt et al. 2011 [46]**	Psychopharmacology (Berl)	ENG	**SPECIFIC** schizophrenia; ICD-10 F20.x	**Pharmaceutical** flupentixol **VS** other first- and second-generation antipsychotics	**INDUSTRY** Bayer Vital	CCA	no	**Insurance** TK (Techniker Krankenkasse), AOK Baden-Wuerttemberg, AOK Westfalen-Lippe, AOK Berlin	costs and effects	yes	n = 2890	**payer** statutory health insurance	1 year
**Stargardt et al. 2008 [47]**	J Ment Health Policy Econ	ENG	**SPECIFIC** schizophrenia; ICD-10 F20.0 to F20.9	**Pharmaceutical** atypical **VS** typical antipsychotic treatment for schizophrenia	**INDUSTRY** Janssen Cilag	CCA	no	**Insurance** TK (Techniker Krankenkasse)	costs and effects	yes	n = 3121	**payer** statutory health insurance	1 year

Outcomes	Costs	Summary Measure unit	Sel. Bias considered	Methods for Sel. Bias	Details on Methods	Data linkage	Handling of Uncertainty	Software
mortality; guideline adherence (according to prescriptions), number of disease-related hospitalizations	total costs; medication costs; outpatient costs; inpatient costs; costs of inpatient rehabilitation and cure	**Seperate** survival time; number of prescriptions; total costs per person	yes	propensity score matching combined with exact matching	PS computed by logistic regression; theoretical reasoning for variable selection; exact matching for age, sex, insurance company, at least one CHD diagnosis and cost class; 1:1 nearest neighbor matching without replacement and caliper of 0.02; SDF to evaluate goodness-of-fit	not applicable for external sources; not addressed for linkage of inpatient and outpatient data	no	**SPSS** for propensity scores; **Microsoft SQL** scripts for matching
life years; QALY	inpatient costs; ambulatory costs; rehabilitation costs; diabetes therapy costs; other medication costs; sickness leave	**ICER and seperate** euros per life-year gained; euros per QALY gained	no	none	-	not applicable (model)	univariate sensitivity analysis with different therapy-switching threshold, discount rate of costs and effects, reduction of costs of rosiglitazone, clinical effectiveness of rosiglitazone treatment	not stated
rehospitalization rate; mean hospital bed days; adverse effects	cost of inpatient care; cost of antipsychotic treatment; cost of other pharmaceutical treatment	**Seperate** risk of rehospitalization; days hospitalized; costs in euros	in part	confounding by switching within drug groups considered	to avoid bias through switching withing the two drug groups, analyses focused on patients who did not switch groups	not applicable for external sources; not addressed for linkage of inpatient and outpatient data	no	**SAS 9.1** for statistical analysis
rehospitalization rate; mean hospital bed days; side effects	cost of inpatient care; cost of antipsychotic treatment; cost of other pharmaceutical treatment	**Seperate** number of hospitalizations; days hospitalized; costs in euros	in part	confounding by severity of disease considered	collection of data on prior hospitalizations with a diagnosis of schizophrenia	not applicable for external sources; not addressed for linkage of inpatient and outpatient data	inclusion of a variable for depot use as a dummy and continuous variable, no considerable impact on comparisons	**SAS 9.1** for statistical analysis
rehospitalization rate; hospital bed days with a diagnosis of schizophrenia per year; number of hospitalizations per year; side effects	cost of inpatient care, cost of antipsychotic treatment, cost for other pharmaceuticals	**Seperate** days hospitalized; length of stay; number of stays; costs in euros stratified by severity	in part	confounding by severity of disease considered	collection of data on prior hospitalizations with a diagnosis of schizophrenia	not applicable for external sources; not addressed for linkage of inpatient and outpatient data	no	**SAS 9.1** for statistical analysis

Study	Journal	Language	Disease	Intervention	Funding	Evaluation Type	Type stated	Routine Data (RD) source	Use of RD	Only RD?	Study Size	Perspective	Time Frame of analysis
**Steinke et al. 2016 [48]**	Acta Derm Venereol	ENG	**SPECIFIC** notalgia paraesthetica (NP) and brachioradial pruritus (BRP) (forms of neuropathic pruritus)	**Pharmaceutical** Capsaicin 8% dermal patch **VS** routine care	**INDUSTRY** Astellas Pharma GmbH	CEA	yes	**Hospital** Center for Chronic Pruritus, Münster	not specified	no	n = 44	**payer** statutory health insurance	6 months
**Stock et al. 2010 [49]**	Health Aff (Millwood)	ENG	**SPECIFIC** type 2 diabetes mellitus (T2DM)	**HCP** DMP **VS** routine care	**NOT SPECIFIED**	CCA	no	**Insurance** Barmer Ersatzkasse	costs and effects	yes	n = 39764	**not stated**	4 years
**Walter et al. 2016 [50]**	Value in Health	ENG	**SPECIFIC** patients with a coronary catheterization	**MP** vascular closing device **VS** manual compression after diagnostic/interventional catherization	**PUBLIC** German Federal Ministry of Education and Research	CCA	no	**Hospital** University Hospital Tübingen	costs and effects	yes	n = 8665	**service provider** hospital	time period of hospital stay

Outcomes	Costs	Summary Measure unit	Sel. Bias considered	Methods for Sel. Bias	Details on Methods	Data linkage	Handling of Uncertainty	Software
pruritus intensity; anxiety and depression; Patient Benefit Index score	cost of inpatient treatment; outpatient treatment; medication; travel and other expeses of patient; costs for loss of time for skin care; indirect costs as loss of productivity	**ICER and seperate** euros per benefit difference	in part	pre/post design	-	not addressed	confidence intervals	**SPSS 22.0** for statistical analysis
mortality rates; major complications; average duration of hospital stay; average number of hospitalizations	drug costs; hospital costs	**Seperate** number and rate of mortality; costs in US dollars	yes	propensity score matching	stepwise logistic regression; a priori selection of variables; 1:1 matching	not applicable for external sources; not addressed for linkage of inpatient and outpatient data	no	**SAS 9.2**
absolute and relative frequencies of complications; unadjusted realtive risk of complications; length of stay	hospital costs	**Seperate** unadjusted RR in percent; costs in euros	yes	regression analysis	regression analysis to adjust length of stay and total costs; covariate adjustment using propensity score (not propensity score matching)	not applicable	confidence intervals	**SAS 9.3** for statistical analysis; **MS Excel** and **PowerPoint** for tables and figures

CCA = cost-consequences analysis; **CEA** = cost-effectiveness analysis; **CUA** = cost-utility analysis; **DALY** = disability-adjusted life years; **DDD** = defined daily dose; **ENG** = English; **GER** = German; **HCP** = health care program; **ICD-10-GM** = international classification of diseases revision 10 German modification; **ICER** = incremental cost-effectiveness ratio; **MP** = medical product or procedure; **NMB** = net monetary benefit; **QALY** = quality-adjusted life years; **QoT** = quarter of treatment; **SGB** = code of social law; **VS** = versus; **WTP** = willingness to pay
